# Patients want more information after surgery: a prospective audit of satisfaction with perioperative information in lung cancer surgery

**DOI:** 10.1186/s13019-018-0707-8

**Published:** 2018-02-01

**Authors:** Nicola Oswald, John Hardman, Amy Kerr, Ehab Bishay, Richard Steyn, Pala Rajesh, Maninder Kalkat, Babu Naidu

**Affiliations:** 1 Institute of Inflammation and Ageing, University of Birmingham Laboratories, University of Birmingham, Queen Elizabeth Hospital Birmingham, Edgbaston, Birmingham, B15 2TT UK; 2 Department of Thoracic Surgery, Heart of England NHS Foundation Trust, Bordesley Green East, Birmingham Heartlands Hospital, Birmingham, B9 5SS UK

**Keywords:** Education, Patient, Lung neoplasm, Assessment, Patient outcome, Patient satisfaction, Thoracic surgery

## Abstract

**Background:**

Receiving information about their disease and treatment is very important to patients with cancer. There is an association between feeling appropriately informed and better quality of life. This audit aimed to estimate patient satisfaction with perioperative information in those undergoing surgery for lung cancer and any change in satisfaction over time.

**Methods:**

A questionnaire (EORTC-Info-25) was administered prospectively to patients preoperatively and up to six months postoperatively. The preoperative questionnaire was completed by 292 patients and 88 free text comments were completed. Intrapersonal responses were compared over time.

**Results:**

Patients were highly satisfied with information prior to surgery. The overall helpfulness of information did not change over time but satisfaction with the amount of information decreased. Patients who received more information about ‘the disease’ and ‘things you can do to help yourself get well’ were less likely to report a drop in satisfaction (Odds Ratio 0.858, 95% Confidence interval 0.765 to 0.961, *p* = 0.008 and OR 0.102, 95% CI 0.018 to 0.590, *p* = 0.011 respectively). Free text responses revealed patients most frequently wanted more information on the disease, aftercare and self-care. Suffering complications from surgery was not associated with a change in satisfaction with information postoperatively.

**Conclusions:**

Patients want to know more about their diagnosis, but also how to recover and cope with issues once they have gone home after surgery. Postoperative satisfaction with information may improve if patients are given more information on these topics.

## Background

Cancer patients want to know as much as possible about their illness [1]. Appropriate provision of information is associated with superior health related quality of life, lower levels of anxiety and lower levels of depression amongst cancer survivors [2, 3]. Appropriate information has also been shown to have benefits with regards to pain and preparedness for surgery [3–5]. However, clinic appointments are time limited and there is often a short interval between this appointment and surgery being undertaken [1, 6]. The aim of this audit was to record patient satisfaction with perioperative information and to identify unmet informational needs in patients undergoing lung cancer resection in order to benchmark and then improve our service.

## Methods

A prospective single centre audit was undertaken at a tertiary thoracic surgical unit after registration with the local Audit and Governance department. Consecutive patients attending the preoperative assessment clinic in preparation for any lung resection, pleural procedures or lung cancer staging procedures under general anaesthetic were invited to complete questionnaires. As part of routine care prior to undergoing surgery patients receive information from their Cancer Specialist Nurse, Respiratory Physician, Thoracic Surgeon and Preoperative Assessment Nurse. The audit institution routinely provides a lung surgery handbook and DVD to patients via their Cancer Specialist Nurse.

Preoperative questionnaires were completed in the preoperative assessment clinic. Follow up questionnaires were posted to patients between at six weeks postoperatively, and again at five months postoperatively to assess the performance of the department throughout the surgical pathway. Data on postoperative complications were collected prospectively including the occurrence of atrial fibrillation, wound infection, discharge with a drain in situ, discharge with a urinary catheter in situ, chest infection, the need to have a chest drain reinserted postoperatively, unplanned admission to the Intensive Care Unit, return to theatre and readmission to hospital within 30 days of discharge.

Satisfaction was assessed via administration of the European Organisation for Research and Treatment of Cancer Quality of Life Questionnaire – Information Module (EORTC-QLQ-Info25), which has been validated internationally among cancer patients [7]. The questionnaire multi-item scales are organised across four groups—information about the disease, medical tests, treatment and other services, and eight single items. Scaling of responses between 0% and 100% was performed as per EORTC guidance [8]. Questions about the amount of information (question 52) and helpfulness of information (question 55) were used to assess overall satisfaction.

Data were not normally distributed and so non parametric tests were employed. Comparison of ordinal data before and after surgery was performed using a Wilcoxon signed rank test. Binary logistic regression was performed with the presence of a drop in overall satisfaction as the dependent variable and satisfaction with each topic of information as the independent variables. Analysis was performed using IBM SPSS version 22 (IBM Corp. Armonk, NY). A *p* value of less than 0.05 was deemed statistically significant. Missing data were handled as per the EORTC scoring manual; if more than half of the data points for a group were present a mean was calculated [7, 8]. Free text responses were grouped into themes according to broad categories after review by two individuals.

## Results

Over 27 months 292 patients filled in a response to the preoperative questionnaire, the response rate was 36.5%. Postoperative follow up questionnaires were completed by 85 patients at five months; seven patients did not undergo surgery and 36 died prior to completing a postoperative questionnaire giving a follow up rate of 34.1%. Individual preoperative questionnaires were 95.5% complete, individual postoperative questionnaires were 94.6% complete. The baseline characteristics of the patients included within the study are listed in Table 1. The overall incidence of complications was 34%.

**Table 1:** Baseline patient characteristics

	Total sample (*n* = 292)
Mean age (range)	67 (24–88)
Gender % male	55.5% (162)
Operative procedure	
Cervical mediastinoscopy	4.1% (12)
Wedge resection, segment	18.2% (53)
Metastasectomy	5.5% (16)
Lobectomy/bilobectomy	59.2% (173)
Pneumonectomy	3.4% (10)
Open and close	2.4% (7)
None^a^	2.4% (7)
Other	4.8% (14)
Incision	
VATS	37.3% (109)

^a^Patients did not proceed to surgery for medical reasons

Amongst preoperative questionnaires the highest score was obtained for information about medical tests (median 77.8%, Inter Quartile Range 66.7%–100.0%); the lowest score was recorded for information about other services (median 25.0%, IQR 8.3–50.5%).

**Fig. 1:**
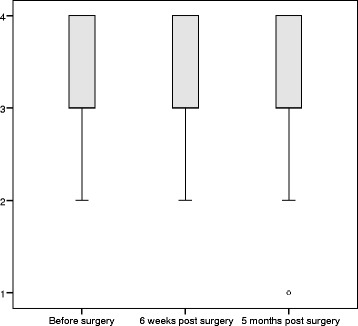
Box plot displaying satisfaction reported in question 55 over time *‘Overall has the information you have received been helpful?’*: 1 = not at all, 2 = a little, 3 = quite a bit, 4 = very much

**Fig. 2:**
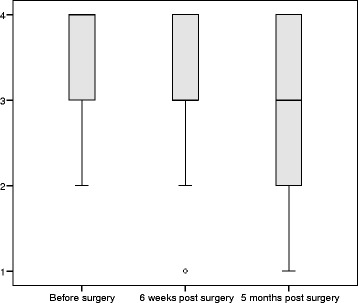
Box plot displaying satisfaction reported in question 52 over time *‘Were you satisfied with the amount of information you received?’*: 1 = not at all, 2 = a little, 3 = quite a bit, 4 = very much

Overall helpfulness of information did not change over time (median 66.7% and IQR 66.7–100% at each time point, *p* = 0.108), however satisfaction with the amount of information was significantly lower postoperatively (*p* = 0.043). Preoperatively the median satisfaction with amount of information was 100% (IQR 66.7–100%), and five months postoperatively the median satisfaction was 66.7% (IQR 33.3–100%). The trends over time are illustrated in Figs. 1 and 2.

The factors underlying the decline in satisfaction postoperatively were investigated further using binary logistic regression and a significant model was found, correctly classifying 84.0% of cases (X^2^ 35.635, *p* < 0.001, Nagelkerke R^2^ 0.683). Patients who received more information about ‘the disease’ and ‘things you can do to help yourself get well’ were less likely to report a drop in satisfaction (Odds Ratio 0.858, 95% Confidence interval 0.765 to 0.961, *p* = 0.008 and OR 0.102, 95% CI 0.018 to 0.590, *p* = 0.011 respectively). Patients who received more information about ‘medical tests’ were more likely to report a drop in satisfaction postoperatively, although this had smaller effect (OR 1.072, 95% CI 1.003–1.146, *p* = 0.041). The presence of a complication did not significantly change satisfaction with information (OR 0.636, 95% CI 0.055 to 7.324, *p* = 0.717).

A total of 88 free text responses were completed by 65 individuals. General comments about information or clinical care were frequent. For example, *‘Cancer Nurse Specialist information was invaluable’*. The topics that patients wished to know more about are shown in Table 2. The most commonly desired topics (the disease, aftercare and self-care) match the topics that were associated with a decline in postoperative satisfaction.

**Table 2:** Topics that patient would like to know more about

Topic	Responses % (n)	Equivalent Info 25 question
Aftercare	17.0% (15)	44, 45, 46, 47
The disease	12.5% (11)	31, 32, 33, 34
Self-care	10.2% (9)	49
Chemo/radiotherapy	6.8% (6)	38
Specific information about their case	6.8% (6)	No question
Prognosis & outcome of surgery	6.8% (6)	32, 34, 39
Everything	5.7% (5)	52
Finances	3.4% (3)	No question
Pain	2.3% (2)	40
The surgery	2.3% (2)	38
Other	26.1% (23)	

The underlying theme of self-care encapsulates what the patient can do to help himself or herself. The underlying theme of aftercare is what healthcare professionals can do to help the patient. There is of course extensive crossover between the two categories with a dynamic interaction between patient and healthcare professional. Within ‘the disease’, four patients wanted to know more about mycobacterium tuberculosis (TB). This is an important consideration for patients undergoing surgery with a diagnostic element because the medical treatment of TB is intensive and may come as a shock to patients. One patient commented that information ‘*made me scared’.*

## Discussion

This report of the findings from a prospective audit of patient satisfaction with perioperative information before and after thoracic surgery is the first to use the EORTC-QLQ-Info25 questionnaire specifically in those undergoing surgery for lung cancer. Satisfaction with information preoperatively was high amongst our patients and compares favourably with published scores [7]. The fall in satisfaction postoperatively may mean patients are less well prepared for surgery than they had thought. This would be consistent with qualitative research in which patients felt prepared for lung cancer surgery preoperatively, but felt less well prepared postoperatively [9]. The qualitative report included follow up limited to five days after surgery, thus our audit covering the weeks and months following discharge provides important additional information that this effect is not limited to the immediate inpatient period. In addition, the quantitative questionnaire used can be completed in any surgical centre to compare and reflect upon the services being provided locally. The decline postoperatively may indicate patients thought they were well informed but with the benefit of hindsight they needed more information preoperatively. Alternatively patients may be well informed before surgery but less well informed during the weeks and months following surgery as they recover. Both interpretations indicate that patients want more information about recovery and carrying on with life after surgery. Research should seek to describe the experience of patients in recovering from surgery after discharge, including the return to normal life, and then the impact of including this information in patient information.

Our finding that complications had no effect on satisfaction with information supports the concept that Patient Reported Outcome Measures (PROMs) measure a different aspect of care to traditional measures such as morbidity and mortality. This is in agreement with a published case-matched analysis that found complications did not impact upon patients’ satisfaction with the quality of care they received [10, 11]. Assessment of quality in surgical lung cancer care currently relates to mortality and resection rate. This audit supports the stance that PROMs are not a surrogate marker of conventional outcomes, but they represent complementary measures with value in their own right.

The consequences of receiving insufficient information may go beyond low patient satisfaction. Patients may have a decreased quality of life for many years following surgery for lung cancer, the reasons for this are currently uncertain [12]. In patients with head and neck cancer, satisfaction with information has been shown to predict postoperative depression and psychological components of quality of life [13]. A variety of formats of information are effective for patient education, resulting in reduced anxiety and increased satisfaction [14, 15]. Patients report wanting to talk with healthcare professionals prior to surgery and then read information independently postoperatively [9]. Patients undergoing thoracic surgery and their carers also frequently use the internet to seek more information [9, 16]. Websites allow the provision of written and audiovisual information that is readily accessible, accurate websites could be a valuable addition to preoperative information.

Our audit findings are subject to some limitations, most importantly the number of missing postoperative questionnaires. However, the patients who did or did not return postoperative questionnaires had similar baseline characteristics and the distribution of operative procedures was similar to national figures [17]. Patients commented upon the large number of questions in each questionnaire during the audit, this may well have contributed to our loss to follow up. A PROM tool which has fewer questions may be more acceptable to patients.

Confounding factors may be present among our patientd including personality types and educational level. Distressed personality types report lower satisfaction and that they have been given less information and those with higher educational levels report lower satisfaction [18, 19]. Intrapersonal comparison should reduce the impact of these in comparing responses across time; in addition the fact that free text responses and binary regression analysis identified similar topics of importance supports the findings being real and not due to a statistical phenomenon.

## Conclusions

Patients feel well informed before lung cancer resection. In the weeks and months following surgery patients feel less well informed and want to know more about the disease they have, recovery and how to cope with issues once they have gone home after surgery. Improving the information given to patients may have additional benefits in terms of quality of life outcomes so research into the patient experience of recovery from lung cancer resection and the impact of including this information in patient information literature is desirable. The findings of this audit were used to develop content for a patient information website available at http://www.thoracicsurgery.co.uk.
